# Supplementary material for: Galloping Bubbles
Source: Nat Commun. 2025 Feb 12;16:1572. doi: 10.1038/s41467-025-56611-5 (PMC11822036; doi:10.1038/s41467-025-56611-5)
Supplement: Supplementary file 1 — Supplementary Information [file 41467_2025_56611_MOESM1_ESM.pdf]

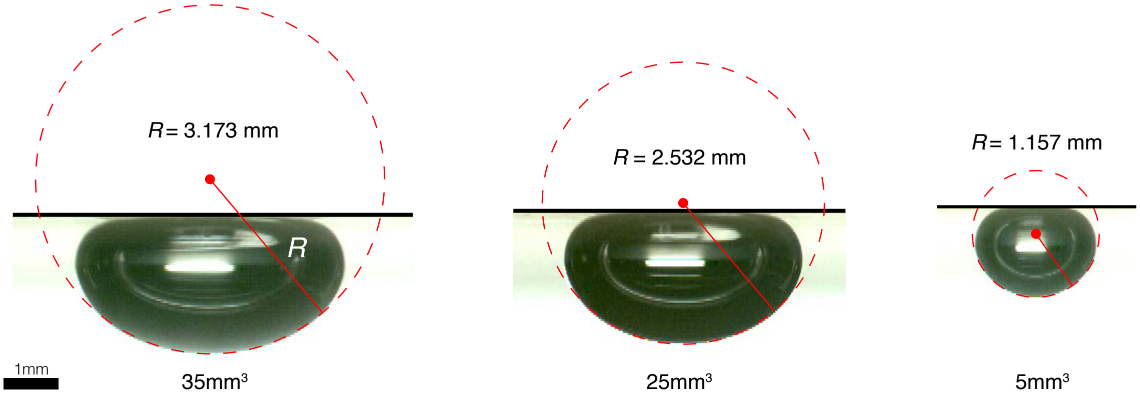

Supplementary Fig. S1. **Bubble equilibrium shapes.** In the absence of vertical vibration ($A = 0$), the equilibrium shape of a bubble with volume $V_b = 25\,\mathrm{mm}^3$ is closest to a hemisphere; a circle can be fitted around the bubble interface with its centre coinciding with the solid-liquid interface. For smaller bubbles, $V_b = 5\,\mathrm{mm}^3$, the base shape is closer to a sphere as surface tension becomes more dominant. Conversely, large bubbles, $V_b = 35\,\mathrm{mm}^3$, become flattened by gravity.

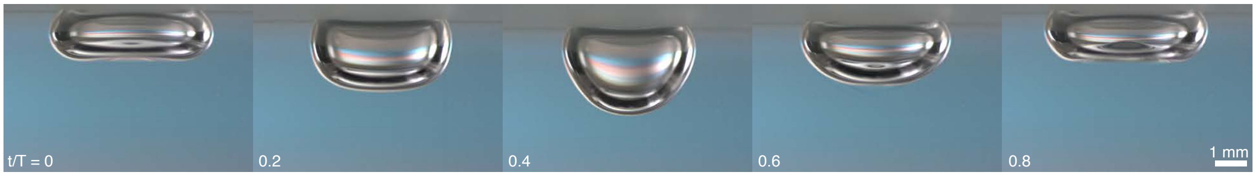

Supplementary Fig. S2. **Symmetric oscillations below the galloping threshold.** The 25 mm^3 bubble shown in Fig. 1a vibrating below the galloping threshold, $A < A_G$, exhibits shape oscillations that are symmetric about the vertical axis. In this regime, the bubble does not self-propel.

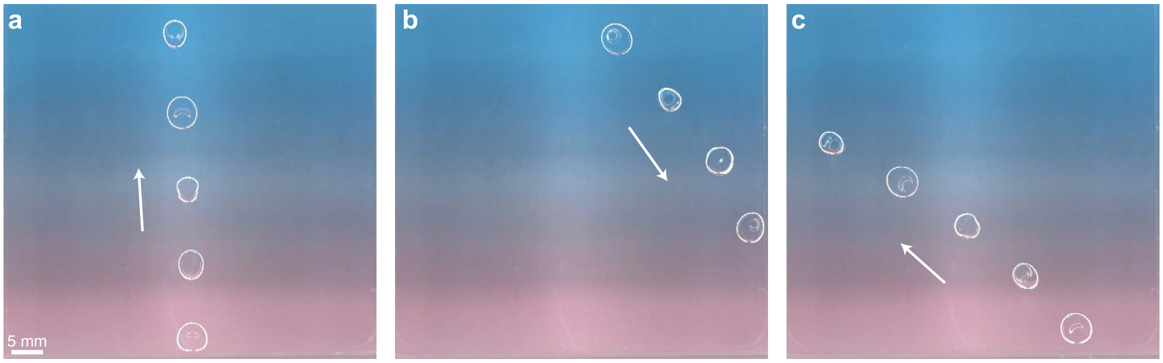

Supplementary Fig. S3. **Bubble motion away from vertical walls.** In the experiments shown in Fig. 1b, the galloping bubble follows a circular trajectory due to the influence of a gentle slope that allows to redirect the bubble without significantly influencing its speed. Away from the walls, the same bubble may travel following straight paths as shown by composite images in(**a-c**). In each image, the time increment is $25T$.

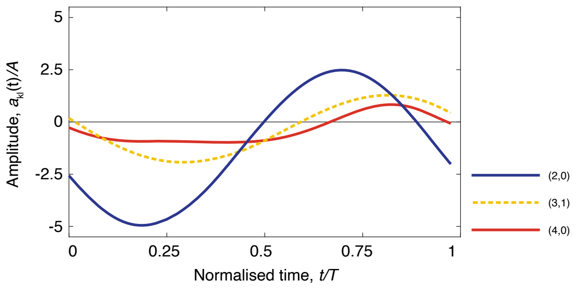

Supplementary Fig. S4. **Instantaneous mode amplitude.** Instantaneous amplitude (normalised by the bath displacement) over an oscillation period for the three dominant vibration modes arising in the hemispherical bubble presented in Fig. 2g with $We = 42$ and $A/R = 0.12$.

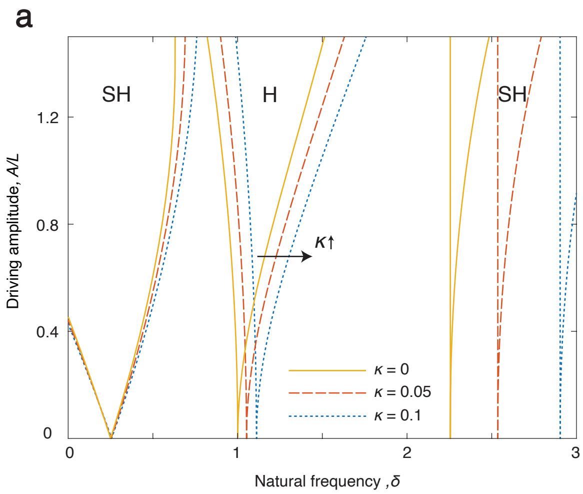

Supplementary Fig. S5. **Stability diagram for the weakly deformable pendulum.** Dependence of the neutral stability curves on the dimensionless driving, $\varepsilon = A/L$, and natural frequency, δ, delineating the transition to harmonic (H) and subharmonic (SH) unstable regimes for a vertically vibrating pendulum (**a**) in the absence of damping, $\gamma = 0$, and (**b**) with damping, $\gamma = 0.02$. The critical threshold rises with the damping, having a more pronounced influence on higher harmonics. As the deformability κ increases, the unstable tongues shift to higher frequencies.

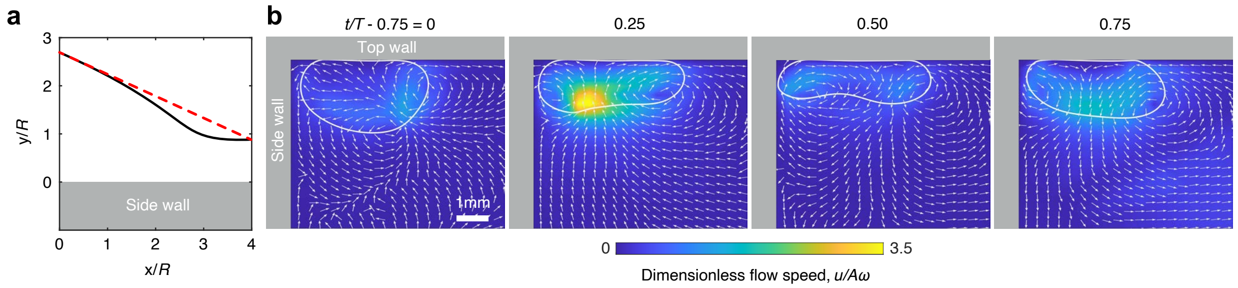

Supplementary Fig. S6. **Attraction to vertical walls.** (**a**) Top view illustrating the trajectory of a simulated galloping bubble (black curve) approaching a vertical side wall (gray area), compared to its original rectilinear path (red line). Here, $V_b = 30\text{mm}^3$, $We = 66.8$, $A/R = 0.14$, and the no-slip condition is applied along the solid boundary. As the separation between the bubble and the wall becomes comparable to the bubble's size, the bubble trajectory deviates from its initial path due to an attractive fluid-mediated interaction with the wall. Along the vertical wall, the galloping speed is 40% greater than in the absence of lateral boundaries, a feature also observed in experiments. (**b**) Lateral view showing a vertical cross-section perpendicular to the side wall, and through the bubble centroid. In this perspective, the bubble gallops towards the viewpoint. Additional asymmetries arise in the interface and flows: the stroke pushing the bubble into the wall is stronger than the one away from it, which holds the bubble against the junction line between the side and top walls. This inherent attraction to lateral walls underpins the bubble's self-sorting and maze-navigation capabilities illustrated in Fig. 5.

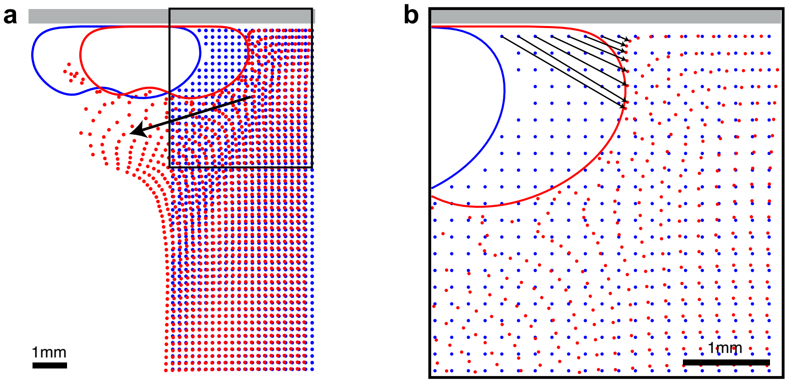

Supplementary Fig. S7. **Particle removal.** (**a**) A simulated galloping bubble with $V_b = 30\,\mathrm{mm}^3$, $We = 66.8$, and $A/R = 0.11$ advects tracer particles from the front over ten oscillation periods, with blue representing $t = 0$ and red representing $t = 10T$. Particles are pushed backward and downward beneath the bubble due to the asymmetry of the bubble's stroke. (**b**) Inset showing particles near the wall being swept downward by the flows induced by the bubble's oscillations. The black arrows indicate the initial and final positions of the particles closest to the wall.
