# Supplementary material for: Galloping Bubbles
Source: Nat Commun. 2025 Feb 12;16:1572. doi: 10.1038/s41467-025-56611-5 (PMC11822036; doi:10.1038/s41467-025-56611-5)
Supplement: Supplementary file 2 — Description of Additional Supplementary Files [file 41467_2025_56611_MOESM2_ESM.docx]

File Name: Supplementary Video 1

Description: **Galloping bubble.** A 25 mm^3^ bubble vibrated vertically with frequency f = 40 Hz oscillates axisymmetric about the vertical axis when the driving amplitude, A = 0.2 mm is below the galloping threshold, A_G_ > A. Here, Re = 20.5, Bo = 2.9, We = 36.2. When the driving amplitude exceeds the galloping threshold, A= 0.46 mm > A_G_, the symmetry about the vertical axis breaks spontaneously and the bubble starts to self-propel along the upper wall. Here, Re = 46.1, Bo = 2.9, We = 36.2. In open space, a galloping bubble may follow straight paths.

File Name: Supplementary Video 2

Description: **Domain exploration modes.** Galloping bubbles exhibit distinct modes of exploration within the experimental domain. Typically, bubbles travel in relatively straight paths, which may become circular due to the boundary of our experimental set-up (see circular border indicated by white dashed line). At higher driving amplitudes, and depending on their volume, bubbles may follow orbital trajectories or transition to a run-and-tumble motion characterized by erratic, unpredictable sharp turns.

File Name: Supplementary Video 3

Description: **Hemispherical galloping bubbles.** Simulations capture the galloping bubbles examined in our experiments and demonstrate that the same self-propulsion emerges in hemispherical bubbles with freely moving contact lines and a 90˚ contact angle. Here, the bubble volume is V_b_ = 30 mm^3^, and the driving frequency and amplitude are f = 40 Hz and A = 0.3 mm, respectively.

File Name: Supplementary Video 4

Description: **Flow fields.** Side view videos showing the instantaneous flow field around a bubble when the bubble is undergoing symmetric oscillations at low driving amplitude, A = 0.23 mm and as it gallops at higher amplitude, A = 0.31 mm. Here, the bubble volume is V_b_ = 25 mm^3^ and the forcing frequency f = 40 Hz. In both cases, the flows decay away from the bubble over a characteristic length scale of the same order of magnitude as the bubble size, R.

File Name: Supplementary Video 5

Description: **Proof-of-concept applications.** A series of proof-of-concept experiments demonstrate the potential of galloping bubbles for practical applications.

(a) Removal: bubbles formed at a `nucleation’ point depart once they reach a size conducive to triggering the galloping instability (f = 48 Hz, A = 0.32 mm). The two videos show that the bubbles' directions are random.

(b) Size selection: using a syringe pump at a given fixed flow rate (≈ 0.15 mL s^-1^), the size of the bubbles produce may be tuned through the vibrational forcing (V_b_ ≈ 14 mm^3^, f = 48 Hz, A = 0.32 mm for the top video, and V_b_ ≈ 18 mm^3^, f = 55 Hz, A = 0.25 mm for the bottom).

(c) Sorting: composite video showing bubbles of increasing volumes being sorted into collectors of correspondingly increasing size thanks to the tendency of galloping bubbles to follow lateral walls.

(e) Navigation: composite video showing three bubbles of different volumes navigating and eventually solving a fluid maze.

(f) Cleaning: video showing the gradual removal of particles from the top solid surface as a galloping bubble explores the domain following a chaotic run-and-tumble motion.
